# Micronuclei induced by reverse transcriptase inhibitors in mononucleated and binucleated cells as assessed by the cytokinesis-block micronucleus assay

**DOI:** 10.1590/S1415-47572010005000084

**Published:** 2010-12-01

**Authors:** Eloir D. Lourenço, Viviane S. do Amaral, Mauricio Lehmann, Rafael R. Dihl, Virginia M. Schmitt, Kênya S. Cunha, Maria L. Reguly, Heloisa H. R. de Andrade

**Affiliations:** 1 Programa de Pós-Graduação em Diagnóstico Genético e Molecular, Universidade Luterana do Brazil, Canoas, RSBrazil; 2 Laboratório da Toxicidade Genética, Programa de Pós-Graduação em Genética e Toxicologia Aplicada, Universidade Luterana do Brazil, Canoas, RSBrazil; 3 Laboratório de Biologia Molecular, Instituto de Pesquisas Biomédicas, Pontifícia Universidade Católica do Rio Grande do Sul, Porto Alegre, RSBrazil; 4 Laboratório de Genética Toxicológica, Departamento de Bioquímica e Biologia Molecular, Instituto de Ciências Biológicas, Universidade Federal de Goiás, Goiânia, GOBrazil; 5 Laboratório de Estomatologia, Hospital de Clínicas de Porto Alegre, Universidade Federal do Rio Grande do Sul, Porto Alegre, RSBrazil

**Keywords:** lamivudine, micronucleus assay, stavudine, transcriptase inhibitors, zidovudine

## Abstract

This study evaluated the clastogenic and/or aneugenic potential of three nucleoside reverse transcriptase inhibitors (zidovudine - AZT, lamivudine - 3TC and stavudine - d4T) using the cytokinesis-block micronucleus (CBMN) assay in human lymphocyte cultures. All three inhibitors produced a positive response when tested in binucleated cells. The genotoxicity of AZT and 3TC was restricted to binucleated cells since there was no significant increase in the frequency of micronuclei in mononucleated cells. This finding indicated that AZT and 3TC caused chromosomal breakage and that their genotoxicity was related to a clastogenic action. In addition to the positive response observed with d4T in binucleated cells, this drug also increased the frequency of micronuclei in mononucleated cells, indicating clastogenic and aneugenic actions. Since the structural differences between AZT and 3TC and AZT and d4T involve the 3' position in the 2'-deoxyribonucleoside and in an unsaturated 2',3',dideoxyribose, respectively, we suggest that an unsaturated 2', 3', dideoxyribose is responsible for the clastogenic and aneugenic actions of d4T.

Nucleoside reverse transcriptase inhibitors (NRTIs) are widely used to treat human immunodeficiency virus (HIV) infections and prolong the survival of HIV-infected patients. NRTIs are incorporated into viral DNA, leading to blockade of the viral reverse transcriptase nucleotide binding site and the termination of replication (Huang and Jolicoeur, 1990).

Zidovudine (3'-azido-3'-deoxythymidine; AZT), lamivudine (2'-deoxy-3'-thiacytidine; 3TC) and stavudine (2',3'-didehydro2',3'-dideoxythymidine; d4T) are nucleoside reverse transcriptase inhibitors (NRTIs). Typically, AZT and 3TC are administered as part of highly active antiretroviral therapy protocols. The genotoxic manifestations of AZT include mutagenesis, chromosomal aberrations and telomere shortening (Huang and Jolicoeur, 1990; Dobrovolsky *et al.*, 2005). The major mechanisms of mutation induction by AZT involve large interstitial deletions and mitotic recombination (Mittelstaedt *et al.*, 2004).

Based on the mechanism of AZT action, the genotoxicity of NRTIs has been defined as a complex, intricate network of events that may lead to large-scale genomic instability as a result of drug incorporation into DNA (Olivero *et al.*, 1997). Numerous reports based on experiments *in vitro* (cultured cells) and *in vivo* have described the induction of micronuclei by AZT (Gonzalez-Cid and Larripa, 1994; Agarwal and Olivero, 1997; Aruna and Jagetia, 2001; Wutzler and Thust, 2001; Kirsch-Volders *et al.*, 2002; Olivero, 2007; Guimarães *et al.*, 2008). However, only a few studies have investigated the genotoxic potential of 3TC and d4T (Schilling *et al.*, 1995; Von Tungeln *et al.*, 2002; Carter *et al.*, 2007; Kaur and Singh, 2007).

In this study, we investigated the clastogenic and/or aneugenic potential of AZT, 3TC and d4T using the cytokinesis-block micronucleus (CBMN) assay in human lymphocyte cultures. We also examined the structure-activity relationships for the mutagenicity of these compounds. Previous studies suggested that the assessment of micronuclei in mononucleated cells might be an interesting additional parameter in the CBMN assay since these cells can reflect aneugenic effects (Elhajouji *et al.*, 1998; Rosefort *et al.*, 2004). For this reason, we analyzed both mononucleated and binucleated cells in an attempt to detect differences in the responses to these three NRTIs and their spectrum of chromosomal mutagenicity.

Peripheral blood lymphocytes were obtained by venipuncture from two healthy females (26 and 29 years of age, referred to as donors 1 and 2, respectively) and one healthy male (24 years of age, donor 3). All of the donors were non-smokers who had not recently been exposed to significant ionizing radiation or mutagens known to induce micronuclei. None of the donors had any structural or numerical chromosomal alterations in their karyotypes. For each donor, one series of cultures was prepared with two parallel cultures (`duplicates') for every concentration of mutagen tested. Whole blood cultures were done as recommended by Migliore *et al.* (1989). For each culture, 0.8 mL of heparinized blood samples was added to 8 mL of RPMI-1640 medium (Sigma Chemical Co., St. Louis, MO, USA) containing 10% fetal calf serum, 1% penicillin/streptomycin and 80 μL of phytohemaglutinin (10 μL/mL) (PHA, Gibco, New Zealand).

The NRTIs were purchased from IQUEGO (Indústria Química de Goiás, Goiânia, GO, Brazil) and included zidovudine (3'-azido-3'-deoxythymidine or AZT; CAS no. 30516-87-1), lamivudine (2'-deoxy-3'-thiacytidine or 3TC; CAS no. 134678-17-4) and stavudine (2',3'-didehydro2'-,3'-dideoxythymidine or d4T; CAS no. 3056-17-5). Bleomycin (BLM, Blenoxane^®^; Bristol Myers Squibb S.A., São Paulo, SP, Brazil) was used as a positive control. All of the compounds were diluted in sterile distilled water, which was also used as a negative control.

Forty-eight hours before harvesting, the cell cultures were supplemented with NRTIs, BLM or sterile distilled water, all of which were sterilized by filtration through a Sartorius 0.22-mm pore filter. After an additional 44 h, all of the cultures were supplemented with 6 μg of cytochalasin B/mL (cyt-B; Sigma) to prevent cells that had completed one nuclear division from undergoing cytokinesis. The use of cyt-B allowed the accumulation of virtually all dividing cells at the binucleate stage, regardless of their degree of synchrony. The total incubation time for all cultures was 72 h at 37 °C. After incubation, the cells were exposed to a hypotonic solution (0.075 M KCl) for 5 min. This procedure preserved the cytoplasm and allowed the assignment of micronuclei to their corresponding main nucleus. The cells were fixed in methanol:glacial acetic acid (3:1, v/v), spotted onto clean microscope slides, air-dried, and stained with Giemsa stain. Micronucleated cells were analyzed by light microscopy and scored for binucleated and mononucleated lymphocytes, with micronuclei being scored based on standard recognition criteria proposed by Fenech *et al.* (2003). To avoid scorer bias, the slides were coded so as to blind the scorer to the sample. For each culture, 500 binucleated and 500 mononucleated cells were examined. The micronuclei frequencies for the control and NRTI-treated cultures are shown in Table 1. Since there were no significant differences between the data for duplicate cultures these results were pooled to yield 3,000 binucleated and mononucleated cells for each concentration.

The nuclear division index (NDI) was used as a parameter for cytotoxicity. For this, 1,000 cells per donor were screened at 400X magnification to determine the frequency of cells with one, two, three or four nuclei, after which the NDI was calculated according to the formula:



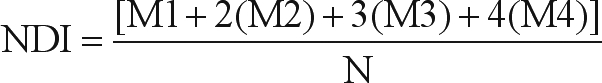


where M1 to M4 represent the number of cells with 1-4 nuclei and N is the total number of cells scored. Since nuclear divisions are asynchronous in CBMN-blocked lymphocytes there may be cells with three nuclei (Eastmond and Tucker, 1989; Rosefort *et al.*, 2004).

The frequencies of binucleated and mononucleated cells with micronuclei in the treated cultures were compared with their respective controls by using one-way analysis of variance (ANOVA) followed by the one-tailed Dunnett *post-hoc* test. The same test was used to study the variability between donors and to compare the NDI of samples and controls. The equality of variances was assessed with Levene's test. A value of p < 0.05 indicated significance.

Table 1 shows that BLM and d4T significantly lowered the NDI values at all concentrations tested. Low NDI values were also observed at the three highest concentrations of AZT and 3TC. All of the NRTIs tested signific±uclei in binucleated cells (p ≤ 0.05). This increase was concentration-dependent for AZT and 3TC, except at the lowest concentration (125 μg/mL). In the case of d4T, the greatest increases in micronucleus frequency occurred at low concentrations and tended to decrease with increasing drug concentration.

AZT and 3TC did not significantly increase the frequency of micronuclei in mononucleated cells at any of the concentrations tested, possibly indicating that these two antiretrovirals are not aneugenic agents. In contrast, the lowest d4T concentration enhanced the frequency of micronuclei whereas the three highest concentrations had the opposite (negative) effect. The latter observation was probably related to the low NDI observed for the three highest d4T concentrations.

The aim of this study was to evaluate the clastogenic and/or aneugenic actions of the antiretroviral agents AZT, 3TC and d4T based on the formation of micronuclei in binucleated and mononucleated human lymphocytes (CBMN). As shown here, AZT and 3TC were genotoxic only in binucleated cells, which indicated that these NRTIs probably caused chromosomal breakage in human cells. AZT has been shown to induce micronuclei in cell cultures and animals (Gonzalez-Cid and Larripa, 1994; Agarwal and Olivero, 1997; Aruna and Jagetia, 2001; Von Tungeln *et al.*, 2007). AZT also has clastogenic effects seen as the formation of micronuclei in human peripheral blood lymphocytes. In contrast, a negative response has been observed in the *Salmonella typhimurium* gene mutation assay, probably because these bacteria lack the enzyme activation required for AZT phosphorylation and activation seen in mammals (Wutzler and Thust, 2001). AZT incorporation into DNA induces mutations in the hypoxanthine-guanine phosphoribosyltransferase (*Hprt*) and thymidine kinase (*Tk*) genes. As previously reported, AZT also induces chromosomal aberrations, sister chromatid exchange (SCE) and telomere shortening in cultured cells (Olivero, 2007). In the wing somatic mutation and recombination test (SMART) both mutagenic and recombinogenic events contribute to the AZT-induced increase in the frequency of mutant clones, with ~15% and 85% of the mutant spots being of mutational and recombinational origin, respectively (Guimarães *et al.*, 2008).

3TC exerts a positive effect in the L5178Y *Tk*^*+/-*^ mouse lymphoma assay (Von Tungeln *et al.*, 2007) and in clastogenicity assays (Physicians' Desk Reference, 2000), although an inconclusive effect was observed on mammalian gene mutation and SCE (Schilling *et al.*, 1995; Physicians' Desk Reference, 2000). Indeed, 3TC has recently been classified as a weak inducer of SCE, chromosomal aberrations, and micronucleus formation in human peripheral lymphocytes *in vitro* (Bayram and Topaktas, 2008).

In addition to increasing the frequency of micronuclei in binucleated cells, d4T also affected mononucleated cells, possibly by causing chromosomal loss. This suggestion was based on a previous study that mincronucleus induction occurred exclusively in binucleated cells and was related to DNA breaks. Aneugenic drugs such as diethylstilbestrol, griseofulvin and vincristine sulphate increase frequencies of micronuclei in mononucleated and binucleated cells, whereas clastogens such as mitomycin C, bleomycin and doxorubicin increase the frequency of micronuclei only in binucleated cells (Migliore *et al.*, 1989; Rosefort *et al.*, 2004).

d4T is not genotoxic and does not cause cell transformation *in vitro* in most screening assays. According to the Physicians' Desk Reference, 2000. Healthcare Ser. 103 (online version). Micromedex Inc.Physicians' Desk Reference (2000), a weak clastogenic response has been observed in human peripheral lymphocytes *in vitro* and in L5178Y cells. However, more recently, 3TC and d4T were identified as inducers of host cell DNA damage and mutations in two reporter genes, *Hprt* and *Tk*, using a cell cloning assay, which means that they may represent a risk of cancer (Carter *et al.*, 2007). We have also observed that 3TC and d4T induce a large incidence of mutant spots in the SMART assay. Indeed, ~86% of the genotoxicity of 3TC and 76% of that of d4T is linked to recombinogenic activities, which means that approximately 14% of the genotoxicity of 3TC and 24% of that of d4T can be imputed to the induction of mutational events (Franchi *et al.*, 2009). Since in the SMART assay only two gene loci may express point mutations, we inferred that the mutagenicity observed was more probably related to chromosomal alterations. However, as far as we know, no aneugenic properties such as those observed here have previously been ascribed to d4T.

**Figure 1:**
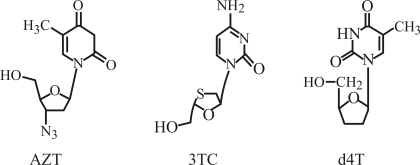
Chemical structure of zidovudine (AZT), lamivudine (3TC) and stavudine (d4T).

Structurally, the major difference among these three NRTIs resides in the 3' position of the 2'-deoxyribonucleoside, where AZT has an azido group, 3TC has a sulfur atom and d4T an unsaturated 2',3',dideoxyribose group (Figure 1). Despite the structural differences between AZT and 3TC, there were no significant divergences in their clastogenic effects, indicating that neither the azido nor the sulfur group interferes in the drugs' genotoxicity. The presence of 2',3',dideoxyribose instead of the azido group or sulfur atom seen in the other molecules ascribed a new property to d4T, namely aneugenic activity. Although scoring mononucleated cells offers a new promise for the detection of micronuclei induced by aneugens, the unknown origin/status of these cells can adversely affect the reliability of the data (Fenech, 2000; Rosefort *et al.*, 2004; Clare *et al.*, 2006). In conclusion, the results of this study indicate the CBMN assay provides as useful means of quantitatively assessing the potential of NRTIs to disturb the human genome. Further analysis of the complexity of antiretroviral genotoxicity should reveal additional mechanisms that will allow the development of protective intervention strategies (Norppa and Falck, 2003; Olivero, 2007).

## Figures and Tables

**Table 1:** Spontaneous and induced frequencies of BNMN and MNMN in lymphocyte cultures treated *in vitro* with different concentrations of AZT, 3TC and d4T.

	Donor 1					Donor 2					Donor 3					Average		
	BNMN^c^		MNMN^c^			BNMN^c^		MNMN^c^			BNMN^c^		MNMN^c^			BNMN^d^	MNMN^d^	NDI^e^
	Rep 1	Rep 2	Rep 1	Rep 2		Rep 1	Rep 2	Rep 1	Rep 2		Rep 1	Rep 2	Rep 1	Rep 2				
AZT																		
NC^a^	2	0	0	0		1	1	0	0		1	1	0	0		2.00 ± 1.26	0.00 ± 0.00	2.03 ± 0.16
125 μg/mL	1	2	0	0		1	2	0	0		2	2	0	0		3.34 ± 1.04	0.00 ± 0.00	1.90 ± 0.09
250 μg/mL	3	3	1	0		4	1	0	0		2	2	1	0		5.00 ± 2.10^**^	0.66 ± 1.04	1.90 ± 0.09
375 μg/mL	3	3	0	0		3	3	0	0		3	4	1	0		6.34 ± 0.82^***^	0.34 ± 0.82	1.82 ± 0.12^**^
500 μg/mL	5	6	1	0		5	3	1	0		4	5	1	0		9.34 ± 2.26^***^	1.00 ± 1.10	1.77 ± 0.08^**^
625 μg/mL	4	5	1	0		5	5	0	1		6	5	1	0		10.00 ± 1.26^***^	1.00 ± 1.10	1.72 ± 0.07^***^

3TC																		
NC^a^	1	0	0	0		0	1	0	0		0	0	0	0		0.66 ± 1.04	0.00 ± 0.00	2.02 ± 0.10
125 μg/mL	1	1	0	0		0	2	0	0		2	1	0	0		2.34 ± 1.50	0.00 ± 0.00	1.88 ± 0.75
250 μg/mL	1	2	0	0		2	1	0	0		3	1	0	0		3.34 ± 1.64^**^	0.00 ± 0.00	1.83 ± 0.10^*^
500 μg/mL	2	3	0	0		2	3	0	0		3	2	0	0		5.00 ± 1.10^***^	0.00 ± 0.00	1.80 ± 0.06^**^
750 μg/mL	4	3	0	1		3	3	0	0		2	3	0	0		6.00 ± 1.26^***^	0.34 ± 0.82	1.73 ± 0.10^***^

d4T																		
NC^a^	2	2	0	1		0	0	0	0		0	1	1	1		1.66 ± 1.96	1.00 ± 1.10	2.08 ± 0.15
125 μg/mL	6	5	3	3		3	5	4	3		3	8	4	2		10.00 ± 3.80^***^	6.34 ± 1.50^***^	1.53 ± 0.08^***^
250 μg/mL	3	4	2	1		3	5	1	2		4	4	0	1		7.66 ± 1.50^***^	2.34 ± 1.50	1.47 ± 0.10^***^
500 μg/mL	4	4	3	1		2	3	1	3		2	2	0	0		5.66 ± 1.96^**^	2.66 ± 2.74	1.47 ± 0.12^***^
750 μg/mL	2	4	1	2		4	2	1	0		2	4	2	1		6.00 ± 2.18^**^	2.34 ± 1.50	1.33 ± 0.08^***^

PC^b^																		
6 μg/mL	16	19	0	1		23	18	1	1		20	18	0	1		37.66 ± 4.80	1.34 ± 1.04	1.80 ± 0.14

^a^NC: negative control; ^b^PC: positive control (bleomycin). ^c^MNMN: mononucleated micronucleated cells; BNMN: binucleated micronucleated cells. For each replicate (Rep), 500 binucleates were scored to give a total of 1,000 binucleated cells (*i.e.* 2,000 nuclei) for each donor. No cells had more than one micronucleus. ^d^Total number of micronuceli in 1,000 binucleates ± standard deviation (SD). ^e^NDI: nuclear division index. ^*^p ≤ 0.05, ^**^p ≤ 0.01 and ^***^p ≤ 0.001 compared to the corresponding negative controls.

## References

[AgarwalandOlivero1997] Agarwal R.P., Olivero O.A. (1997). Genotoxicity and mitochondrial damage in human lymphocytic cells chronically exposed to 3'-azido2', 3'dideoxythymidine. Mutat Res.

[ArunaandJagetia2001] Aruna R., Jagetia G.C. (2001). Azidothymidine induces dose dependent increase in micronuclei formation in cultured HeLa cells. Pharmazie.

[BayramandTopaktas2008] Bayram S., Topaktas M. (2008). Confirmation of the chromosome damaging effects of lamivudine in *in vitro* human peripheral blood lymphocytes. Environ Mol Mutagen.

[Carteretal2007] Carter M.M., Torres S.M., Cook D.L., McCash C.L., Yu M., Walker V.E., Walker D.M. (2007). Relative mutagenic potencies of several nucleoside analogs, alone or in drug pairs, at the *Hprt* and *Tk* loci of human TK6 lymphoblastoid cells. Environ Mol Mutagen.

[Clareetal2006] Clare M.G., Lorenzon G., Akhurst L.C., Marzin D., van Delft J., Montero R., Botta A., Bertens A., Cinelli S., Thybaud V. (2006). SFTG international collaborative study on *in vitro* micronucleus test: II. Using human lymphocytes. Mutat Res.

[Dobrovolskyetal2005] Dobrovolsky V.N., Mcgarrity L.J., Von Tungeln L.S., Mittelstaedt R.A., Morris S.M., Beland F.A., Heflich R.H. (2005). Micronucleated erythrocyte frequency in control and azidothymidine - Treated *Tk+/+, Tk+/-* and *Tk-/-* mice. Mutat Res.

[EastmondandTucker1989] Eastmond D.A., Tucker J.D. (1989). Identification of aneuploidy-inducing agents using cytokinesis-blocked human lymphocytes and an antikinetochore antibody. Environ Mol Mutagen.

[Elhajoujietal1998] Elhajouji A., Cunha M., Kirsch-Volders M. (1998). Spindle poisons can induce polyploidy by mitotic slippage and micronucleate mononucleates in the cytokinesis-block assay. Mutagenesis.

[Fenech2000] Fenech M. (2000). The *in vitro* micronucleus technique. Mutat Res.

[Fenechetal2003] Fenech M., Chang W.P., Kirsch-Volders M., Holland N., Bonassi S., Zeiger E. (2003). HUMN Project: Detailed description of the scoring criteria for cytokinesis-block micronucleus assay using isolated human lymphocyte cultures. Mutat Res.

[Franchietal2009] Franchi L.P., Pentiado N.H.G.R., Silva R.N., Guimarães N.N., Jesuino R.S.A., Andrade H.H.R., Lehmann M., Cunha K.S. (2009). Mutagenic and recombinagenic effects of lamivudine and stavudine antiretrovirals in somatic cells of *Drosophila melanogaster*. Food Chem Toxicol.

[Gonzalez-CidandLarripa1994] Gonzalez-Cid M., Larripa I. (1994). Genotoxic activity of azidothymidine (AZT) in *in vitro* systems. Mutat Res.

[Guimaraesetal2008] Guimarães N.N., Pereira K.C., Andrade H.H.R., Lehmann M., Cunha K.S. (2008). Comparative analysis of genetic toxicity of AZT and ddI anti-retrovirals in somatic cells of *Drosophila**melanogaster*. Environ Mol Mutagen.

[HuangandJolicoeur1990] Huang M., Jolicoeur P. (1990). Characterization of the gag/fusion protein encoded by the defective Duplan retrovirus inducing murine acquired immunodeficiency syndrome. J Virol.

[KaurandSingh2007] Kaur P., Singh R. (2007). *In vivo* interactive effect of garlic oil and vitamin E against stavudine induced genotoxicity in *Mus musculus*. Indian J Exp Biol.

[Kirsch-Voldersetal2002] Kirsch-Volders M., Vanhauwaert A., De Boeck M., Decordier L. (2002). Importance of detecting numerical versus structural chromosome aberrations. Mutat Res.

[Miglioreetal1989] Migliore L., Nieri M., Amodio S., Loprieno N. (1989). The human lymphocyte micronucleus assay: A comparison between whole-blood and separated-lymphocyte cultures. Mutat Res.

[Mittelstaedtetal2004] Mittelstaedt R.A., Von Tungeln L.S., Shaddock J.G., Dobrovolsky V.N., Beland F.A., Heflich R.H. (2004). Analysis of mutations in the *Tk* gene of *Tk*^*+/*^ mice treated as neonates with 3'-azido-3'-deoxythymidine (AZT). Mutat Res.

[NorppaandFalck2003] Norppa H., Falck G.C. (2003). What do human micronuclei contain?. Mutagenesis.

[Olivero2007] Olivero O.A. (2007). Mechanisms of genotoxicity of nucleoside reverse transcriptase inhibitors. Environ Mol Mutagen.

[Oliveroetal1997] Olivero O.A., Anderson L.M., Diwan B.A., Haines D.C., Harbaugh S.W., Moskal T.J., Jones A.B., Rice J.M., Riggs C.W., Logsdon D. (1997). Transplacental effects of 3'-azido-2',3'-dideoxythymidine (AZT): Tumorigenicity in mice and genotoxicity in mice and monkeys. J Natl Cancer Inst.

[PhysiciansDeskReference2000] Physicians' Desk Reference (2000). Eletronic Library Medical Economics Company, Inc.

[Rosefortetal2004] Rosefort C., Fauth E., Zanki H. (2004). Micronuclei induced by aneugens and clastogens in mononucleate and binucleate cells using the cytokinesis block assay. Mutagenesis.

[Schillingetal1995] Schilling B.E., Nelson D.R., Proctor J.E., Diamond S.S., Kaul S., Hawkins H.C. (1995). The nonclinical toxicologic profile of stavudine. Curr Ther Res.

[VonTungelnetal2002] Von Tungeln L.S., Hamilton L.P., Dobrovolsky V.N., Bishop M.E., Shaddock J.G., Heflich R.H., Beland F.A. (2002). Frequency of *Tk* and *Hprt* lymphocyte mutants and bone marrow micronuclei in B6C3F(1)/*Tk+/-* mice treated neonatally with zidovudine and lamivudine. Carcinogenesis.

[VonTungelnetal2007] Von Tungeln L.S., Williams L.D., Doerge D.R., Shaddock J.G., Mcgarrity L.J., Morris S.M., Mittelstaedt R.A., Heflich R.H., Beland F.A. (2007). Transplacental drug transfer and frequency of *Tk* and *Hprt* lymphocyte mutants and peripheral blood micronuclei in mice treated transplacentally with zidovudine and lamivudine. Environ Mol Mutagen.

[WutzlerandThust2001] Wutzler P., Thust A. (2001). Genetic risks of antiviral nucleoside analogues - A survey. Antiviral Res.

